# Gut instincts: vitamin D/vitamin D receptor and microbiome in neurodevelopment disorders

**DOI:** 10.1098/rsob.200063

**Published:** 2020-07-08

**Authors:** Destiny Ogbu, Eric Xia, Jun Sun

**Affiliations:** 1 Division of Gastroenterology and Hepatology, Medicine, University of Illinois at Chicago, Chicago 60612, IL, USA; 2 Marian University College of Osteopathic Medicine, Indianapolis, IN, USA; 3 UIC Cancer Center, Chicago, IL, USA

**Keywords:** neurodevelopmental disorders, gut–brain axis, dysbiosis, microbiome, inflammation, vitamin D

## Abstract

The gut microbiome regulates a relationship with the brain known as the gut–microbiota–brain (GMB) axis. This interaction is influenced by immune cells, microbial metabolites and neurotransmitters. Recent findings show gut dysbiosis is prevalent in autism spectrum disorder (ASD) as well as attention deficit hyperactivity disorder (ADHD). There are previously established negative correlations among vitamin D, vitamin D receptor (VDR) levels and severity of ASD as well as ADHD. Both vitamin D and VDR are known to regulate homeostasis in the brain and the intestinal microbiome. This review summarizes the growing relationship between vitamin D/VDR signalling and the GMB axis in ASD and ADHD. We focus on current publications and summarize the progress of GMB in neurodevelopmental disorders, describe effects and mechanisms of vitamin D/VDR in regulating the microbiome and synoptically highlight the potential applications of targeting vitamin D/VDR signalling in neurodevelopment disorders.

## 1. Introduction

The term ‘microbiome’ refers to the collective genomes of the microbial communities (bacteria, viruses and fungi) in all niches of the human body, whereas ‘microbiota’ refers to the microorganisms living in specific locations, such as the gut microbiota. There has been increasing emphasis on the role of the microbiota in physiology, suggesting that the microbiota can be considered as another ‘human organ’. The gut microbiota is affected by intrinsic (i.e. genetics, age) and extrinsic (i.e. diet, medications) factors [1]. There is emerging evidence that this invisible ‘organ’ is a key driver of human health and disease [2].

The GMB axis describes the bidirectional relationship between the central nervous system (CNS) and gut microbiome, and this relationship is thought to be involved in neurodevelopmental disorders [3]. GMB functions are carried out through immune cell activity, metabolite synthesis and neurotransmitter production [3]. ASD and ADHD could be described as gut–brain disorders due to the potential role of gut microbiota [4]. The fifth edition of the *Diagnostic and Statistical Manual* (DSM-5) defines ASD diagnosis as reduced social-emotional reciprocity and nonverbal communication, whereas ADHD is characterized by hyperactivity and inattentiveness [5].

Vitamin D and VDR have novel functions beyond their classical roles in bone development. VDR activates innate immunity and affects intestinal development patterns. In the adult, vitamin D has regulatory roles in mucosal immunity, host defense and inflammation via VDR. This interaction involves host factors and the gut microbiome [6–11]. Vitamin D/VDR signalling is another pillar supporting the potential role of the GMB axis in the aetiology of ASD and ADHD [12,13]. The purpose of this review is to summarize the progress of GMB in neurodevelopmental disorders, describe roles of vitamin D/VDR in regulating the microbiome, and discuss and highlight the potential role of vitamin D/VDR signalling in the gut–brain–microbiota in ASD and ADHD.

## 2. Gut microbiota in neurodevelopmental disorders

An adult gut is inhabited by approximately 10^13^ to 10^14^ microorganisms, which collectively exceed the number of human cells in the entire body [14]. Beginning at birth, the gut microbiota acts as an organ by influencing other organs through breakdown of nutrients, biosynthesis of metabolites and modulation of immune system [15]. The microbiota can influence the CNS directly or indirectly. Direct connections include its role in tryptophan metabolism resulting in production and release of neuroactive metabolites, such as serotonin, in the systemic circulation. Indirect connections include modulation of CNS inflammation, alteration of nutrient absorption and modification of metabolism of exogenous drugs [16]. Mice models have shown gut microbiota may change in neurological disorders, such as Parkinson's disease [17], Alzheimer's disease [18] and amyotrophic lateral sclerosis [19].

Gut microbiota alterations may be associated with cognitive as well as social deficits. In a cohort of 89 one-year-olds, a relative overabundance of the genus *Bacteroides* was associated with higher scores on Mullen's Early Learning Composite at age two [20]. Investigators examined if gut microbiota from infancy influenced neurodevelopment later (preschool age). In fecal samples collected between ages 3 and 6 months, abundance of the order *Clostridiales* was associated with poorer communication scores in the Ages and Stages Questionnaire 3 (*β*, –1.12; 95% CI, −2.23 to −0.01; *p* = 0.05), personal and social scores (*β*, –1.44; 95% CI, −2.47 to −0.40; *p* = 0.01) at age three [21].

The relationship between GI health and ASD individuals is growing. A longitudinal study of 124 children with ASD and 242 controls revealed that children with ASD had an increased incidence of constipation (*p* = 0.003) compared with controls [22]. In a cohort of 164 individuals with ASD, 49% exhibited common GI abnormalities such as constipation (26%) and diarrhoea (22%) [23]. The mechanism behind GI impairments has not been established. GI irregularities may be caused by microbial dysbiosis which is an imbalance of microbes in the gut microbiota that could alter the integrity of the intestinal barrier [24]. Srikantha *et al.* [3] predicted intestinal permeability caused by a reduction of barrier-forming tight junctions could be a potential biomarker in ASD pathology. However, the mechanism behind this hypothesis is unexplored.

ASD may be induced by maternal immune activation. Meta-analysis of over 40 000 ASD cases in 15 studies found a 1.13 OR (95% CI: 1.03–1.23) increase in ASD risk following maternal infection during pregnancy [25]. Notably, the effects of infection may persist following birth. When measured at age 2–5 years, 97 ASD-diagnosed children born to mothers with infection during pregnancy had the serum inflammatory cytokines IL-1*β*, IL-6, IL-12 and TNF-α elevated by 76%, 72%, 14% and 28%, respectively, compared with healthy controls, with higher levels correlated to worse disease symptoms [26]. The same cytokines are implicated in inflammatory bowel diseases [27]. IL-6 leads to expansion of CD4T-cells leading to chronic inflammation in the gut [27]. IL-1*β* induces production of inflammatory cytokines and promotes further expression of IL-6 by enterocytes [28]. Lastly, IL-12 is the chief cytokine for Th1/CD8T-cell differentiation [29]. Biologics against IL-12 (ustekinumab) and TNF-α (adalimumab) are routinely used to treat inflammatory bowel disease, but their usefulness in altering the course of ASD has not been investigated, probably due to high cost and immunosuppressive side effects [30].

In mice, maternal administration of the viral mimic polyriboinosinic-polyribocytidylic acid (poly(I:C)) induces infection. In these offspring, IL-1*β*, IL-6 and TNF-α are found elevated in the fetal brain and ASD symptoms result [31]. When vitamin D is co-administered with poly(I:C) in mice, ASD-related deficits in social interaction, stereotyped behaviour and emotional learning and memory were abolished. However, there was no change found in concentration of inflammatory cytokines in the brains of mothers or pups, indicating that vitamin D functions through a different pathway [32]. An important interaction that remains to be explored concerns the duelling inflammatory state brought on by dysbiosis and other conditions (e.g. maternal immune activation). For example, an increase in *Clostridium difficile,* a pathogenic microbe [33], in the infant gut has been associated with formula feeding [34]. While most colonization with *C. difficile* remains subclinical, an increase in *C. difficile* composition is associated with atopic conditions in childhood *C. difficile* levels persisted when measured months later. Indeed, breast milk feeding for at least six months is protective against ASD [35]. Vitamin D in breast milk [36] may contribute to gut microbiome growth during infancy. Future work may focus on comparing the relative effect of a spike in inflammation versus sustained dysbiosis because both have been shown to increase the incidence of ASD.

Some growing ideas to reduce GI irregularities and ASD severity including supplementation of probiotics or prebiotics and fecal microbiome transplant (FMT) have been examined in ASD clinical trials [37]. Probiotics are presumably thought to enhance GI health by reducing gut barrier permeability [38]. A probiotic (Vibosome containing *Lactobacillus* and *Bifidobacterium*) was administered to 13 ASD children (ages 3–12 years) to treat GI symptoms for a 19-week trial period. The Vibosome treatment showed significant improvement in GI complaints (*p* = 0.02) [39]. Another possible solution for ASD-GI comorbidities could be administration of vitamin D, because ASD individuals are often dietarily deficient [12]. The potential of vitamin D to improve behavioural ASD symptoms has been established. A clinical trial of vitamin D supplementation (2000 IU per day) in ASD children (*n* = 42; ages 2.5–8 years) for 12 months found that supplementation reduced behavioural irritability (*p* = 0.01) [40]. However, to our knowledge, no study has specifically examined the GI effects of vitamin D supplementation in ASD individuals. Prebiotic supplementation studies in ASD-GI comorbidities are growing. ASD children (*n* = 30) were given a prebiotic supplementation (B-GOS) to investigate the influence on stool and bowel movement as well as social behaviour scores in ASD individuals. The data were not reported, and researchers report no trend of GI discomfort reductions following B-GOS intervention [41]. A growing public interest in both pre- and probiotics led to a randomized clinical trial that investigated the effect of a probiotic containing *Bifidobacterium infantis* plus prebiotic bovine colostrum product (BCP) mixture in ASD children (*n* = 8; aged 3.9–10.9 years old) with constipation, diarrhoea and/or irritable bowel syndrome. Participants were treated with BCP only and the combination mixture of *B. infantis* plus BCP for 12 weeks. Overall, 75% (6/8) reported greater GI improvement with BCP only treatment compared with 25% (2/8) improvement via combination treatment. A reduced frequency of diarrhoea (BCP: *p* = 0.021 versus combination: *p* = 0.021) and normal stool consistency (BCP: *p* = 0.042 versus combination: *p* = 0.015) was observed in ASD individuals [42]. An open-label clinical trial assessed if FMT from healthy controls (*n* = 20) to ASD-diagnosed children (*n* = 18) rectified constipation, diarrhoea, indigestion and abdominal pain in ASD individuals for 18 weeks. After daily maintenance doses for seven to eight weeks, the study found that 80% children with ASD had decreased GI symptoms (*p* < 0.001) and reduced ASD severity (*p* = 0.002) [43]. A follow-up of this cohort 2 years later showed that treatment subjects saw further improvement of GI and ASD symptoms [44]. The FMT treatment had a long-term effect compared with probiotic and prebiotic treatments. The solution for ASD-GI treatments will keep expanding.

Ming *et al.* hypothesized that altered microbiome composition and increased GI dysfunction may be present in ADHD children [45]. The link between ADHD and gut microbiota is still growing and the current results are varied. The gut microbiota of 14 male ADHD patients (mean age: 11.9 years) and 17 male controls (mean age: 13.1 years) were analysed via next generation 16S rDNA sequencing and examined for diversity and biomarkers. Microbial (α) diversity was significantly decreased (*p*_Shannon_ = 0.036) in ADHD patients compared with controls while β diversity varied between patients and controls (*p*_ANOSIM_ = 0.033, *p*_ADONIS_ = 0.006, *p*_beta_disper = 0.002). At the family level, Bacteroidaceae was overabundant in ADHD patients. The authors suggested the genus *Neisseria* and elevated levels of *Bacteroides spec*. could be associated with juvenile ADHD [46]. External validity is limited by the small sample size in the Prehn-Kristensen study. A different clinical study found that the genus *Bifidobacterium* was abundant (*p* = 0.034) in the gut of ADHD individuals (*n* = 19) compared with healthy controls (*n* = 77). Additionally, a predicted enzyme involved in the synthesis of a dopamine precursor (phenylalanine), cyclohexadienyl dehydratase (CDT) was significantly increased (*p* = 0.038) in ADHD patients compared with controls. *Bifidobacterium* abundance may contribute to the observed differences of CDT in a multiple regression analysis (*p* < 0.001). This finding suggests a hallmark of ADHD, diminished neural reward anticipation (a known functional target of dopamine), may be correlated with overabundance of *Bifidobacterium* in the gut which was positively associated with elevated CDT levels [47].

Studies regarding gut microbiome rebalance in ADHD are limited. A 10-week pilot study investigated the effects of a probiotic mixture (vitamins, minerals, amino acids and antioxidants) on fecal microbiome content in diagnosed ADHD children (*n* = 17; aged 7–12 years). There was no variability among treatment group and placebo in an ADHD scale (ADHD-IV-RS). The treatment group had significantly higher α diversity (*p* = 0.005) compared with controls, changes in Actinobacteria (*W* = 6, clr f statistic = 7.5), reduced *Bifidobacterium* (adj *p* < 0.05) and an inverse relationship between *Bifidobacterium* and ADHD-IV-RS (*p* = 0.04) [48]. These results are promising yet a salient bias is the small sample size. Additionally, the finding raises the question, could one probiotic be more influential in the mixture? Vitamin D as cholecalciferol (200 IU) was included in probiotic mixture. This finding supports the influence of *Bifidobacterium* involvement in ADHD as well as probiotics as a possible treatment.

ADHD is also associated with inflammation dysregulation. In comparison to studies regarding ASD, many fewer studies have been performed correlating maternal infection and ADHD. However, one large cohort study found maternal genitourinary infection was associated with an increase in risk for ADHD (OR = 1.29). Pre-eclampsia was also implicated (OR = 1.19), and both together conveyed highest risk (OR = 1.53) [49]. Comparing a cohort of children with diagnosed ADHD, children with mothers suffering infection displayed more severe ADHD symptoms than those with unstressed or healthy mothers [50]. However, the mechanism of this increased risk is not the same as in ASD, as studies have not identified a specific, definitive correlation between inflammation and ADHD [51–53]. Maternal smoking (pooled RR = 1.58) and obesity (OR = 1.62) are the most widely acknowledged external links to ADHD [54]. It is vital to note that genetics plays a profound role in the pathogenesis of ADHD, with heritability estimated at over 75% [55]. It is interesting to consider whether the gut microbiota of individuals who are predisposed to ADHD, but show no symptoms, may be protective. The ideal study would sample the microbiota of non-ADHD children of parents with ADHD, but does not exist. In fact, very few studies of ADHD include parents with ADHD, possibly because widespread recognition and pharmaceutical treatment of ADHD largely began in the last 25–30 years [56]. Fetal alcohol syndrome, caused by maternal alcohol use *in utero*, may produce an ADHD-like phenotype [57]. Neonates born to mothers who drank any alcohol had a 2.5-fold increase in risk of newborn infection and a 3.4-fold increase in risk if the mother drank heavily [58]. We were unable to find a study linking fetal alcohol syndrome and the microbiome. As adults with ADHD continue to have children, investigation of the protective effects of the gut microbiota on ADHD risk will become more possible.

## 3. Vitamin D/vitamin D receptor signalling in neurodevelopmental disorders

The human body intakes vitamin D as cholecalciferol through fortified dairy and oily fish or through the conversion of 7-dehydrocholesterol by ultraviolet light [59]. Once in the body, pro-vitamin D is twice hydroxylated into active 1,25-dihydroxy vitamin D (1,25(OH)_2_, D_3_), and binds to the VDR in the cytoplasm. Bound VDR then associates with the retinoid X receptor, and the entire complex enters the nucleus to function as a transcription factor. The greater than 900 DNA sequences modulated by the VDR complex are called vitamin D response elements (VDRE) [8]. Notably, vitamin D is known to upregulate the expression of its own receptor [60,61]. VDR functions as a transcription factor [62]. Target genes of VDR include anti-microbial peptide [17] cathelicidin precursor (also called LL-37) [63,64], β-defensin, [64] and the 1, 25(OH) _2_D_3_-regulated VDR-specific, Cyp24 hydroxylase gene. Indeed, approximately 3% of the mouse and human genomes are regulated directly or indirectly by the vitamin D endocrine system, further supporting the possibility of widespread effects of vitamin D and VDR in disease mechanisms [65–67]. In the brain, vitamin D/VDR signalling promotes neuroprotection by increasing intracellular levels of the antioxidants glutathione and superoxide dismutase [68]. Mice models have shown VDR regulates intestinal homeostasis and microbiota by maintaining butyrate-producing bacteria [69], inhibiting inflammation through the autophagy regulator gene ATG16L1 and increasing anti-microbial peptides [70], and regulating intestinal permeability [71]. Furthermore, human *Vdr* gene variation shapes gut microbiome and abundance of Parabacteroides affected by the VDR signalling in both human and mouse samples [72].

ASD has been negatively correlated with maternal serum vitamin D concentration through many phases of development. Meta-analysis of eight studies found that an increase in autism-related traits and diagnosed ASD was correlated with decreased maternal serum vitamin D concentrations. Reductions in cognitive ability were most associated with low concentration in early-mid pregnancy [73]. This association appears to weaken later in pregnancy; a cohort of 4229 Dutch mother–child pairs found that low vitamin D status at mid-gestation and at delivery was associated with ASD symptoms but low vitamin D at delivery alone was not [74]. A cohort of 468 Indian mothers found no correlation between ASD symptoms serum vitamin D measured at 30 ± 2 weeks gestation [75]. These data support the idea that the major role of vitamin D changes throughout pregnancy. vitamin D is required for calcium metabolism, and its role in bone development is greatest during calcification of the fetal skeleton during the 3rd trimester [76]. Maternal vitamin D is drained during pregnancy and rebounds slowly unless supplemented. Adequate vitamin D appears most critical early in pregnancy, which may explain the increased risk (14.4% versus 6.8%) of ASD recurrence in birth intervals of < 18 months versus greater than 4 years between siblings [77].

Although correlation of maternal vitamin D concentration with ASD may diminish through third trimester, the molecule regains foremost importance during the early years of life. In the infant, serum vitamin D has been negatively correlated with ASD diagnosis [78] and ASD severity, and vitamin D concentration is lower in ASD siblings compared with neurologically normal siblings [79]. A clinical trial found that 85 children with ASD had an increase (*p* = 0.041) in *fok1*, a VDR polymorphism, compared with 82 healthy controls. ASD individuals had decreased serum vitamin D levels which support the notion that VDR activity is strongly tied to serum vitamin D levels in ASD patients [80]. Vitamin D supplementation significantly improves ASD symptoms in deficient patients [78,81]. Comprehensive meta-analyses support findings of individual researchers across ethnicities and nationalities [82]. Thus, the correlation between low serum vitamin D and ASD is established, and further studies are best directed toward describing GMB axis.

The observed effect of maternal serum vitamin D concentration is varied in ADHD. In a cohort study, which stratified maternal serum vitamin D into high (greater than 50.7 nmol l^−1^) and low (less than 38.4 nmol l^−1^) groups, the high concentration group showed a reduced incidence of hyperactivity–impulsivity symptoms (IRR = 0.63, 95% CI = 0.39–0.99) and total ADHD-like symptoms (IRR = 0.60, 95% CI = 0.37–0.95) when observed at age 4 [83]. A cohort of 1650 mother–child pairs found an 11% decrease in total ADHD-like symptoms for each 10 ng ml^−1^ increase in serum vitamin D at age 4–5 years [84]. In a different experiment, researchers measured the serum VDR levels of 80 children (40 ADHD diagnosed and 40 healthy controls) ranging from 6–12 years old. Serum VDR levels were significantly decreased (pless than 0.001) in ADHD children compared with controls (1.69 ± 0.22 ng ml^−1^ versus 2.08 ± 0.42 ng ml^−1^, respectively) [85]. On the other hand, a longitudinal study of 965 pairs found no association between serum vitamin D and diagnosed ADHD when offspring were followed from birth to 21 years of age [86], and a comparison of cord blood from 202 ADHD-diagnosed patients and healthy controls found no correlation of vitamin D concentration [87]. The tentative consensus is that maternal serum vitamin D is associated with ADHD-like behavioural issues at young ages but not with the clinical diagnosis of ADHD.

A stronger correlation exists between low serum vitamin D in the growing child and ADHD. Meta-analysis of available studies showed that concentration decreases of 6.75 ng ml^−1^ from average has a 2.57 OR (95% CI = 1.09–6.04). Similarly, prospective studies show that low perinatal vitamin D concentrations increase risk of diagnosis later in life (RR: 1.40; 95% CI = 1.09–1.81). This meta-analysis must be interpreted with caution, as removal of one study abolishes the correlation [88]. Considering the risk of ASD and ADHD observed with vitamin D deficiency, vitamin D may join folate as recommended supplementation in women preparing for pregnancy. In a different experiment, researchers measured the serum VDR levels of 80 children (40 ADHD diagnosed and 40 healthy controls) ranging from 6 to 12 years old. Serum VDR levels were significantly decreased (*p* < 0.001) in ADHD children compared with controls (1.69 ± 0.22 ng ml^−1^ versus 2.08 ± 0.42 ng ml^−1^, respectively). The results for these experiments suggest ADHD may have an effect on VDR levels.

## 4. Gut microbiota and neurotransmitters in autism spectrum disorder and attention deficit hyperactivity disorder

Gut microbiota controls neurobehaviour via modulating brain insulin sensitivity and metabolism of tryptophan, the precursor of serotonin [89]. Increased influx of tryptophan into the brain by HFD could be related to increased blood insulin levels. The neurotransmitter serotonin is low in the brain of ASD individuals [90]. Positron emission tomography scans of autistic children (average age 6.6 years) and their non-autistic siblings (average age 9.9 years) revealed asymmetric changes in serotonin production in the frontal cortex and thalamus of the autistic children [91]. Serotonin synthesis is limited by the enzymes tryptophan hydroxylase 1 (TPH1) in the periphery and TPH2 in the brain and enteric nervous system. The balance of TPH1/2 is under transcriptional control by a VDRE. Vitamin D upregulates transcription of TPH2 and downregulates transcription of TPH1, suggesting that vitamin D deficiency may contribute to lower serotonin levels in the brain [90]. In vitro, 24 h culture of glioblastoma, HCT-116 and HEK-293 cells with vitamin D produced dose-dependent upregulation of TPH2 transcription [92]. In rats, vitamin D supplementation after birth caused a dose-dependent increase in TPH2 expression in the prefrontal cortex [93]. Lastly, vitamin D supplementation in rats represses the transcription of serotonin transporter and monoamine oxidase mRNA and therefore raises serotonin levels in the brain by modulating both production and breakdown [94].

The BALB/c mice containing a loss-of-function mutation in TPH2 showed a 20% decrease in TPH2 mRNA and 28% fewer TPH2 immunolabelled neurons compared with TPH2 wild-type C57BL/6 J mice [95]. Russo *et al* [96] found that BALB/c mice quantitatively exhibited reduced social behaviour and increased anxious behaviour compared with C57BL/6 J mice. Intriguingly, this study found that in either strain, TPH2 activity was not significantly correlated to the changes in sociability or anxiety. Finally, complete TPH2 knockout in mice generates a psychologic phenotype characteristic of ASD [97]. To our knowledge, no *in vivo* experiment linking maternal vitamin D and offspring TPH1/2 expression has been attempted.

TPH2 is also responsible for producing serotonin in the enteric nervous system (ENS) in the gut. Serotonergic neurons are the first to develop in the ENS where they direct the patterning of future neurons and neurotransmitter secretors [98]. TPH2 knockout mice show intestinal dysmotility. Serotonin dysregulation, secondary to vitamin D deficiency, has been linked to Inflammatory bowel syndrome [41] and inflammatory bowel disease [99,100]. It is possible that serotonin is the common mediator in the changes seen in each of these conditions. Overall, the effect of vitamin D in the neurotransmitter axis of ASD is underexplored, and a secondary process via the microbiome remains plausible.

A hypothesis in the axis of ADHD is alteration of dopamine (DA) and norepinephrine function. DA functions in emotional response, reward, motivation, motor activity and attention, while norepinephrine is a an adrenergic neurotransmitter activating the sympathetic nervous system [101]. Decreased dopamine function may be caused by decreased dopamine release, lower density of dopaminergic neurons or lower levels by each neuron, or overly rapid clearance at the site of action. *In vitro*, adding norepinephrine to culture of rat mesencephalic cells increased the differentiation of dopaminergic neurons [102].

Following release into the synaptic cleft, dopamine activity is terminated via breakdown by monoamine oxidase [103] or via reuptake into the presynapse by the dopamine transporter (DAT) or into synaptic vesicles by vesicular monoamine transporter 2 (VMAT2) [104]. The main described pathway of ADHD involves overabundance of DAT resulting in diminished duration and intensity of DA action [105]. The ADHD medication methylphenidate increases DA activity by binding and inhibiting DAT, while mixed amphetamine salts mainly inhibit VMAT2. Understanding of ADHD in the context of the GMB axis and VDR is limited but growing. In rats, maternal vitamin D deficiency was associated with increased DAT density in the nucleus accumbens of female, but not male, offspring [106]. A study of 96 children with ADHD found that vitamin D supplementation (50 000IU/week) for eight weeks improved ADHD symptoms as assessed by the Conners Parent Rating Scale [107,108]. Vitamin D supplementation produced an increase in dopamine, but not serotonin, in children with ADHD [108].

Norepinephrine is a second neurotransmitter implicated in ADHD pathogenesis. Analogous to the perturbation seen with DA, overactivity of the norepinephrine transporter (NET) causes rapid reuptake of NE into the presynaptic cytoplasm [109]. An alternative ADHD treatment, amphetamine, increases the activity of dopamine and norepinephrine in the brain by displacing them from synaptic vesicles and into the synaptic clefts [110]. Although the simplicity of dopamine as the chief neurotransmitter in reward and motivation in ADHD has been challenged, the dopamine/NE theory remains an acceptable mechanism because of the effectiveness of amphetamine medication [111,112]. Studies have indicated that up to two-thirds of individuals with ADHD have a comorbid mental disorder, such as mood or anxiety disorder, which may be used as a starting point to study ADHD [113].

In ADHD, alterations in neurodevelopment are evident *in utero*. Rats born to vitamin D-deficient mothers display grossly increased lateral ventricle volume and altered appearance of the dopaminergic substantia nigra [114]. VDR activation drives the expression of tyrosine hydroxylase, the rate limiting step in dopamine production [115]. In rats, this activation takes effect *in utero* between E12 and E15 which supports the correlation of maternal vitamin D deficiency in ADHD [116]. DAT overexpression is still believed to be the primary axis, but the effect of low serum vitamin D is certainly strong enough to potentiate or exacerbate the problem. Future work in the neurotransmission axis of ADHD may need to be creative and novel in the incorporation of genetics into experimental conditions.

## 5. Microbial metabolite in autism spectrum disorder and attention deficit hyperactivity disorder

In humans, metabolites such as short-chain fatty acids (SCFA) are produced via bacterial fermentation in the colon [117], in contrast with mice where fermentation of dietary carbohydrate takes place in the cecum [118]. SCFA consists of 2–6 carbon chains which positively alter the gut microbiome by enhancing anti-inflammatory processes and regulating the enteric neuroendocrine system to promote gut homeostasis [4]. Adequate production of SCFAs has shown positive effects in various diseases including obesity, diabetes, inflammatory bowel diseases as well as psychiatric and neurologic disorders, which has become an interesting aspect of GMB interactions [119]. Metabolome profiling in ASD and ADHD cases is ongoing via clinical trials and mouse models.

Propionate is the SCFAs most produced by ASD prevalent microorganisms [120,121]. SCFA may exert influence on the CNS by binding to free fatty acid receptors [107] in the brain. Propionate bound to the receptor FFAR3 on human brain entholieum inhibited pathways associated with non-specific microbial infections via a CD14-dependent mechanism, suppressed expression of LRP-1 and protected the BBB from oxidative stress via NRF2 signalling [122]. This study was done in post-mortem brains. The influence of SCFAs on a normal functioning brain is unknown. By contrast, butyrate levels in the brain are not naturally high enough to modulate histone deacetylase inhibition in the gut [123].

In order to understand gut microbiome contributions in developing ASD, researchers transplanted microbiota from ASD human donors to germ-free mice and found ASD microbiota may induce hallmark autistic behaviours. Mice colonized with microbiota from ASD human donors had a different microbial composition: a significant decrease in *Bacteroidetes*, *Bacteroides* and *Parabacteroides,* with an increase in *Akkermansia, Sutterella* and *Lachnospiraceae*. The brains of mice colonized with ASD-associated microbiota display alternative splicing of ASD-relevant genes. Metabolome profiles of mice harbouring ASD microbiota (*n* = 20; 4–7 mice per donor) show distinct metabolites in the colon may modulate ASD behaviour. The metabolites taurine and 5-aminovaleric acid (5AV) (*p* = 0.0243) were significantly reduced in mice harbouring ASD microbiota compared with control germ-free mice. Metabolome profile differences between mice models may result from microbial metabolism [124]. This study suggests ASD causes alterations in distinct bacterial phylum, which may lead to the production of certain SCFAs. Taurine is essential for brain development [125], whereas 5AV is described as an anticonvulsant in mice [126]. Clinical trials regarding endogenous SCFA levels in ASD and ADHD are still being developed.

Multiple clinical trials found variation in fecal SCFA within ASD individuals. A clinical trial comparing the levels of fecal SCFAs between ASD children (*n* = 23) and neurotypical children (*n* = 31) aged 11–12 years old found ASD children had higher levels of total SCFAs (136.6 ± 8.7 versus 111.1 ± 6.6 mmol kg^−1^) compared with neurotypical controls. Specifically, ASD individuals had higher concentrations of acetate, propionate, butyrate, isobutyrate, valerate and isovalerate compared with neurotypical children. Researchers concluded higher concentrations of fecal SCFAs in ASD may not be disastrous to the health of ASD participants [127]. In fact, the high concentration in this cohort was not due to different diets. Additionally, a different clinical assessment of fecal SCFAs in ASD individuals found ASD individuals (*n* = 23) had significantly higher levels of isopropanol (*p* = 0.022) in stool compared with healthy controls (*n* = 21) individuals aged 4–17 years old. High levels of isopropanol were hypothesized to cause GI disturbances like abdominal pain [128]. The microbes *Clostridium beijerinckii* and *C. aurantibutyricum* convert acetone to isopropanol [129,130] yet none of the listed microbes where detected in the feces of ASD or neurotypical children [128]. Isopropanol is rapidly absorbed throught the GI tract [131] yet isoproponal poisioning irritates mucosal surfaces and causes GI impairment such as abdomal pain [132]. Isopropanol is also an organic solvent that preserves fecal SCFA [133]. A 2019 study analysed the relationship between gut microbiome and fecal SCFAs in Chinese autistic and neurotypical children. The presence of SCFAs was altered in ASD individuals; acetic and butyrate levels decreased while valeric acid was increased in the ASD group [134]. A recent study compared fecal SCFA levels between an ASD cohort (*n* = 26) and healthy control (*n* = 24) and found significantly reduced levels of acetate, propionate and butyrate in ASD individuals [135].

Regardless of the variability of SCFA in the previous studies, propionic acidaemia (PA) at birth is suggested to increase ASD risk. PA is a propionate deficiency caused by reduced propionyl-CoA carboxylase (PCC) activity in the liver and other tissues. Interestingly, PA and ASD share most of their core symptoms and multiple case studies report ASD as a comorbidity to PA [136–138]. However, only a few ASD cases were associated with PA: PA has been reported in a 7-year-old girl with ASD [139], five ASD patients [137] and four patients with abnormal PCC and ASD [138]. Overall, the relationship between SCFA and ASD phenotypes is still growing. Differentiation in SCFA concentration suggests the importance to investigate other factors that alter SCFA concentration within ASD.

The SCFA levels associated with ADHD pathogenesis is largely unknown due to difficulty in identifying definite biomarkers. A pilot study investigated microbial differences in the microbiome between ADHD and neurotypical adolescent boys. The investigators found a significant difference between control and ADHD microbial composition. They also found a relative overabundance of *Bifidobacterium* predicts ADHD by upregulating synthesis of phenylalanine, a precursor of dopamine, and decreases neural reward anticipation [47]. A potential biomarker includes intermediate products of tryptophan such as kynurenine, kynurenic acid (KA) and xanthurenic acid (XA) [6]. These metabolites may influence the immune system and neurotransmission in ADHD via inflammatory pathways [140]. VDR deficiency could enhance kynurenine metabolite levels, which may be implicated in ADHD pathology, yet the results are inconclusive. A Norwegian study compared serum levels of kynurenines in 133 adult ADHD patients versus adult controls (18–40 years) and found the ADHD group did not have lower levels of tryptophan, kynurenic acid or xanthurenic acid [141]. These findings contradict a Roman study testing serum kynurenine metabolite in ADHD children (*n* = 102), who exhibited increased kynurenine (+48.6%) and reduced serum KA (−11.2%) and XA levels (−12.5%) compared with healthy controls (*n* = 62) [142]. Limitations in both studies include age, location and serum level measures, which showed kynurenine metabolite activity throughout the body instead of the CNS; both studies only analysed serum levels in ADHD patients. Assessment of intestinal VDR in ADHD microbiome may highlight VDR influence in ADHD metabolites. Unlike ASD, the complexity of ADHD is hard to recapitulate in a mouse model. One study proposed a that mice lacking *Fez1*, a gene in the nervous system, which leads to hyperactivity and impulsivity phenotypes [143]. This mouse model has common ADHD phenotypes, yet some areas are unclear. The authors did not assess the gut microbiota of *Fez1-KO* mice. Furthermore, ADHD studies in metabolites are limited. It would be novel to identify the types and levels of metabolites in ADHD individuals.

Our research has shown that loss of intestinal epithelial VDR leads to an increase in butyrate-producing bacteria in intestinal inflammation [70]. It may be further implicated in the GMB axis of neurologic disorders. Deletion of intestinal epithelial VDR increased kynurenine, a pathway associated with inflammatory neurological disorder in a mouse model [144]. Our recent study has also shown that the mice with VDR deletion in immune cells displayed significant downregulation of quinolinate and tocopherol pathway-derived metabolites and increase in nicotinamide. Quinolinate acts as a neurotoxin, pro-inflammatory mediator and prooxidant molecule. These changes indicate a potential role of VDR in neurophysiology. The role of vitamin D/VDR in gut–brain axis needs further investigation in future research [144].

## 6. Concluding remarks

**Figure 1.**
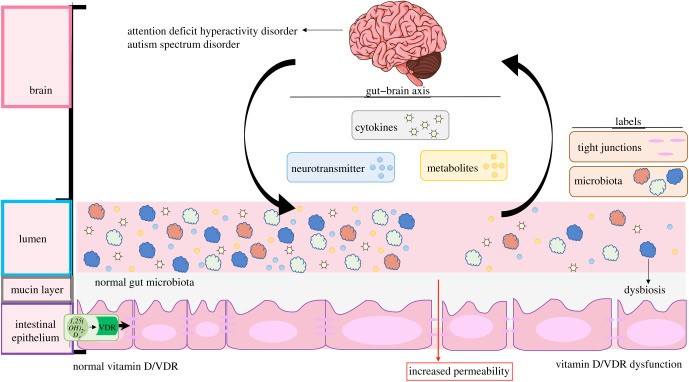
A working model of the potential roles of vitamin D/VDR signalling in ASD and ADHD via the GMB. Vitamin D intake improved GI deficits in ASD individuals yet the mechanisms in vitamin D/VDR signalling are unexplored. Susceptibility to ASD is correlated with polymorphisms in the *VDR* gene as well as low vitamin D in the serum. VDR may decrease microbial dysbiosis, enhance tight junction proteins as well as increase serotonin production, alleviate pro-inflammatory cytokines and increase commensal SCFA production via the GMB.

ASD is characterized by a multitude of social deficits while ADHD is characterized by inattentiveness, impulsivity and hyperactivity [5]. However, changes in social and behavioural impairments overtime create a predicament in identifying definite biomarkers for both disorders. Gut microbiome disorders are observed in ASD and ADHD. Thus, microbial composition may be an effective biomarker for ASD and ADHD. Our studies and others have demonstrated that VDR regulates the gut microbiome by inhibiting inflammation, maintaining barrier functions and promoting microbial homeostasis [70–72]. Susceptibility to ASD is correlated with polymorphisms in the *VDR* gene as well as low vitamin D in the serum. It is still unclear if reduction of vitamin D/VDR signalling in the intestinal lumen contributes to the low microbial diversity seen in the gut of ASD and ADHD individuals. We speculate that VDR may modify gut microbiota in ASD and ADHD through GMB axis, such as cytokines, neurotransmitters and SCFAs (figure 1).

Gut microbial patterns in ASD individuals show decreased microbial diversity and some GI irregularities. Vitamin D supplementation reduced irritability in ASD individuals, yet the influence on GI dysfunction and microbial composition is limited. Additionally, the influence of vitamin D supplements via intestinal VDR should be explored to understand how VDR exerts influence in ASD. Immune activity in genetic ASD resembles immune activity in inflammatory bowel disease yet the influence of VDR in ASD immune activity is lacking. VDR may alter neurotransmitter activity in ASD via transcriptional regulation of tryptophan metabolism, the precursor of serotonin, yet the implications of VDR in serotonin activity are limited. Additionally, metabolites enhanced by VDR loss are unexplored in ASD cases. Fecal SCFAs concentrations are altered in ASD individuals yet inconsistencies among studies, suggest that factors, such as diet, age, location and/or cohort size may contribute to the observed differences. Characterization of abnormal SCFAs within ASD warrants further investigation.

There are indirect relationships linking VDR and ADHD via GMB axis. Activation of VDR drives the expression of a rate limiting step in dopamine production, tyrosine hydroxylase. VDR deficiency enhances tryptophan metabolites which are pronounced in the serum of some ADHD individuals. The gut microbiota in ADHD revealed an overabundance of *Bifidobacterium* which acts as a functional target for dopamine. Its influence on the GI barrier is unexplored.

Understanding of gut microbiota involvement in ASD and ADHD is growing. It is highly unlikely that the breadth of effects of vitamin D/VDR dysregulation is limited to just these two disorders. The interplay between brain development, gut microbiota and the VDR may be implicated in other CNS diseases.

## Data accessibility

This article has no additional data.

## References

[1] O'Hara AM, Shanahan F 2006 The gut flora as a forgotten organ. EMBO Rep. 7, 688–693. (10.1038/sj.embor.7400731) 16819463 PMC1500832

[2] Belkaid Y, Harrison OJ 2017 Homeostatic immunity and the microbiota. Immunity 46, 562–576. (10.1016/j.immuni.2017.04.008) 28423337 PMC5604871

[3] Srikantha P, Mohajeri MH 2019 The possible role of the microbiota-gut-brain axis in autism spectrum disorder. Int. J. Mol. Sci. 20, 2115. (10.3390/ijms20092115) PMC6539237 31035684

[4] Kelly JR, Minuto C, Cryan JF, Clarke G, Dinan TG 2017 Cross talk: the microbiota and neurodevelopmental disorders. Front. Neurosci. 11, 490 (10.3389/fnins.2017.00490) 28966571 PMC5605633

[5] Association AP 2013 Diagnostic and statistical manual of mental disorders, 5th edn Arlington, VA: American Psychiatric Association.

[6] Lloyd-Price J et al 2019 Multi-omics of the gut microbial ecosystem in inflammatory bowel diseases. Nature 569, 655–662. (10.1038/s41586-019-1237-9) 31142855 PMC6650278

[7] Bakke D, Sun J 2018 Ancient nuclear receptor VDR with new functions: microbiome and inflammation. Inflamm Bowel Dis. 24, 1149–1154. (10.1093/ibd/izy092) 29718408 PMC6148749

[8] Bakke D, Chatterjee I, Agrawal A, Dai Y, Sun J 2018 Regulation of microbiota by vitamin D receptor: a nuclear weapon in metabolic diseases. Nucl. Receptor Res. 5, 101377 (10.11131/2018/101377) 30828578 PMC6392192

[9] Barbachano A, Fernandez-Barral A, Ferrer-Mayorga G, Costales-Carrera A, Larriba MJ, Munoz A 2017 The endocrine vitamin D system in the gut. Mol. Cell Endocrinol. 453, 79–87. (10.1016/j.mce.2016.11.028) 27913273

[10] Del Pinto R, Ferri C, Cominelli F 2017 Vitamin D axis in inflammatory bowel diseases: role, current uses and future perspectives. Int. J. Mol. Sci. 18, 2360 (10.3390/ijms18112360) PMC5713329 29112157

[11] Dimitrov V, White JH 2017 Vitamin D signaling in intestinal innate immunity and homeostasis. Mol. Cell Endocrinol. 453, 68–78. (10.1016/j.mce.2017.04.010) 28412519

[12] Cannell JJ 2017 Vitamin D and autism, what's new? Rev. Endocr. Metab. Disord. 18, 183–193. (10.1007/s11154-017-9409-0) 28217829

[13] Petra AI, Panagiotidou S, Hatziagelaki E, Stewart JM, Conti P, Theoharides TC 2015 Gut-microbiota-brain axis and its effect on neuropsychiatric disorders with suspected immune dysregulation. Clin Ther. 37, 984–995. (10.1016/j.clinthera.2015.04.002) 26046241 PMC4458706

[14] Gilbert JA, Blaser MJ, Caporaso JG, Jansson JK, Lynch SV, Knight R 2018 Current understanding of the human microbiome. Nat. Med. 24, 392–400. (10.1038/nm.4517) 29634682 PMC7043356

[15] Turroni F et al 2020 The infant gut microbiome as a microbial organ influencing host well-being. Ital. J. Pediatr. 46, 16 (10.1186/s13052-020-0781-0) 32024556 PMC7003403

[16] Cryan JF et al 2019 The microbiota-gut-brain axis. Physiol. Rev. 99, 1877–2013. (10.1152/physrev.00018.2018) 31460832

[17] Sampson TR et al 2016 Gut microbiota regulate motor deficits and neuroinflammation in a model of Parkinson's disease. Cell. 167, 1469–1480. (10.1016/j.cell.2016.11.018) 27912057 PMC5718049

[18] Zhang L et al 2017 Altered gut microbiota in a mouse model of Alzheimer's disease. J. Alzheimers Dis. 60, 1241–1257. (10.3233/JAD-170020) 29036812

[19] Wu S, Yi J, Zhang YG, Zhou J, Sun J 2015 Leaky intestine and impaired microbiome in an amyotrophic lateral sclerosis mouse model. Physiol. Rep. 3, e12356. 25847918 10.14814/phy2.12356 PMC4425962

[20] Carlson AL et al 2018 Infant gut microbiome associated with cognitive development. Biol. Psychiatry. 83, 148–159. (10.1016/j.biopsych.2017.06.021) 28793975 PMC5724966

[21] Sordillo JE et al. 2019 Association of the infant gut microbiome with early childhood neurodevelopmental outcomes: an ancillary study to the VDAART randomized clinical trial. JAMA Netw. Open. 2, e190905 (10.1001/jamanetworkopen.2019.0905) 30901046 PMC6583279

[22] Ibrahim SH, Voigt RG, Katusic SK, Weaver AL, Barbaresi WJ 2009 Incidence of gastrointestinal symptoms in children with autism: a population-based study. Pediatrics 124, 680–686. (10.1542/peds.2008-2933) 19651585 PMC2747040

[23] Kang V, Wagner GC, Ming X 2014 Gastrointestinal dysfunction in children with autism spectrum disorders. Autism Res. 7, 501–506. (10.1002/aur.1386) 24753336

[24] Sun J 2010 Vitamin D and mucosal immune function. Curr. Opin. Gastroenterol. 26, 591–595. (10.1097/MOG.0b013e32833d4b9f) 20639756 PMC2955835

[25] Jiang HY, Xu LL, Shao L, Xia RM, Yu ZH, Ling ZX, Yang F, Deng M, Ruan B 2016 Maternal infection during pregnancy and risk of autism spectrum disorders: a systematic review and meta-analysis. Brain Behav. Immun. 58, 165–172. (10.1016/j.bbi.2016.06.005) 27287966

[26] Ashwood P, Krakowiak P, Hertz-Picciotto I, Hansen R, Pessah I, Van de Water J. 2011 Elevated plasma cytokines in autism spectrum disorders provide evidence of immune dysfunction and are associated with impaired behavioral outcome. Brain Behav. Immun. 25, 40–45. (10.1016/j.bbi.2010.08.003) 20705131 PMC2991432

[27] Atreya R, Neurath MF 2005 Involvement of IL-6 in the pathogenesis of inflammatory bowel disease and colon cancer. Clin. Rev. Allergy Immunol. 28, 187–196. (10.1385/CRIAI:28:3:187) 16129903

[28] Moon MR, Parikh AA, Pritts TA, Kane C, Fischer JE, Salzman AL, Hasselgren P-O 2000 Interleukin-1beta induces complement component C3 and IL-6 production at the basolateral and apical membranes in a human intestinal epithelial cell line. Shock. 13, 374–378. (10.1097/00024382-200005000-00005) 10807012

[29] Pearce EL, Shen H 2007 Generation of CD8T cell memory is regulated by IL-12. J. Immunol. 179, 2074–2081. (10.4049/jimmunol.179.4.2074) 17675465

[30] Teng MW, Bowman EP, McElwee JJ, Smyth MJ, Casanova JL, Cooper AM, Cua DJ 2015 IL-12 and IL-23 cytokines: from discovery to targeted therapies for immune-mediated inflammatory diseases. Nat. Med. 21, 719–729. (10.1038/nm.3895) 26121196

[31] Schwartzer JJ, Careaga M, Onore CE, Rushakoff JA, Berman RF, Ashwood P 2013 Maternal immune activation and strain specific interactions in the development of autism-like behaviors in mice. Transl. Psychiatry. 3, e240 (10.1038/tp.2013.16) 23481627 PMC3625915

[32] Vuillermot S, Luan W, Meyer U, Eyles D 2017 Vitamin D treatment during pregnancy prevents autism-related phenotypes in a mouse model of maternal immune activation. Mol. Autism. 8, 9 (10.1186/s13229-017-0125-0) 28316773 PMC5351212

[33] Penders J, Stobberingh EE, van den Brandt PA, Thijs C 2007 The role of the intestinal microbiota in the development of atopic disorders. Allergy 62, 1223–1236. (10.1111/j.1398-9995.2007.01462.x) 17711557

[34] Azad MB et al 2013 Gut microbiota of healthy Canadian infants: profiles by mode of delivery and infant diet at 4 months. CMAJ. 185, 385–394. (10.1503/cmaj.121189) 23401405 PMC3602254

[35] Schultz ST, Klonoff-Cohen HS, Wingard DL, Akshoomoff NA, Macera CA, Ji M, Ji M, Bacher C 2006 Breastfeeding, infant formula supplementation, and Autistic Disorder: the results of a parent survey. Int. Breastfeed J. 1, 16 (10.1186/1746-4358-1-16) 16978397 PMC1578554

[36] Ballard O, Morrow AL 2013 Human milk composition: nutrients and bioactive factors. Pediatr. Clin. North Am. 60, 49–74. (10.1016/j.pcl.2012.10.002) 23178060 PMC3586783

[37] Jandhyala SM, Talukdar R, Subramanyam C, Vuyyuru H, Sasikala M, Nageshwar Reddy D 2015 Role of the normal gut microbiota. World J. Gastroenterol. 21, 8787–8803. (10.3748/wjg.v21.i29.8787) 26269668 PMC4528021

[38] Ng QX, Loke W, Venkatanarayanan N, Lim DY, Soh AYS, Yeo WS 2019 A systematic review of the role of prebiotics and probiotics in autism spectrum disorders. Medicina 55, 129. 10.3390/medicina55050129 PMC6571640 31083360

[39] Arnold LE et al 2019 Probiotics for gastrointestinal symptoms and quality of life in autism: a placebo-controlled pilot trial. J. Child Adolesc. Psychopharmacol. 29, 659–669. (10.1089/cap.2018.0156) 31478755 PMC7364307

[40] Mazahery H et al 2019 A randomised controlled trial of vitamin D and ω-3 long chain polyunsaturated fatty acids in the treatment of irritability and hyperactivity among children with autism spectrum disorder. J. Steroid Biochem. Mol. Biol. 187, 9–16. (10.1016/j.jsbmb.2018.10.017) 30744880

[41] Grimaldi R et al 2018 A prebiotic intervention study in children with autism spectrum disorders (ASDs). Microbiome. 6, 133 (10.1186/s40168-018-0523-3) 30071894 PMC6091020

[42] Sanctuary MR et al 2019 Pilot study of probiotic/colostrum supplementation on gut function in children with autism and gastrointestinal symptoms. PLoS ONE 14, e0210064 (10.1371/journal.pone.0210064) 30625189 PMC6326569

[43] Kang DW et al 2017 Microbiota transfer therapy alters gut ecosystem and improves gastrointestinal and autism symptoms: an open-label study. Microbiome. 5, 10 (10.1186/s40168-016-0225-7) 28122648 PMC5264285

[44] Kang DW et al 2019 Long-term benefit of microbiota transfer therapy on autism symptoms and gut microbiota. Sci Rep. 9, 5821 (10.1038/s41598-019-42183-0) 30967657 PMC6456593

[45] Ming X, Chen N, Ray C, Brewer G, Kornitzer J, Steer RA 2018 A gut feeling: a hypothesis of the role of the microbiome in attention-deficit/hyperactivity disorders. Child Neurol. Open 5, 2329048X18786799 (10.1177/2329048X18786799) PMC6047248 30023407

[46] Prehn-Kristensen A et al 2018 Reduced microbiome α diversity in young patients with ADHD. PLoS ONE 13, e0200728 (10.1371/journal.pone.0200728) 30001426 PMC6042771

[47] Aarts E et al 2017 Gut microbiome in ADHD and its relation to neural reward anticipation. PLoS ONE 12, e0183509 (10.1371/journal.pone.0183509) 28863139 PMC5581161

[48] Stevens AJ, Purcell RV, Darling KA, Eggleston MJF, Kennedy MA, Rucklidge JJ 2019 Human gut microbiome changes during a 10 week randomised control trial for micronutrient supplementation in children with attention deficit hyperactivity disorder. Sci. Rep. 9,10 128 (10.1038/s41598-019-46146-3) 31300667 PMC6625977

[49] Mann JR, McDermott S 2011 Are maternal genitourinary infection and pre-eclampsia associated with ADHD in school-aged children? J. Atten. Disord. 15, 667–673. (10.1177/1087054710370566) 20837984

[50] Grizenko N, Shayan YR, Polotskaia A, Ter-Stepanian M, Joober R 2008 Relation of maternal stress during pregnancy to symptom severity and response to treatment in children with ADHD. J. Psychiatry Neurosci. 33, 10–16. 18197267 PMC2186370

[51] Corominas-Roso M, Armario A, Palomar G, Corrales M, Carrasco J, Richarte V, Ferrer R, Casas M, Ramos-Quiroga JA 2017 IL-6 and TNF-α in unmedicated adults with ADHD: relationship to cortisol awakening response. Psychoneuroendocrinology. 79, 67–73. (10.1016/j.psyneuen.2017.02.017) 28262601

[52] Oades RD, Myint AM, Dauvermann MR, Schimmelmann BG, Schwarz MJ 2010 Attention-deficit hyperactivity disorder (ADHD) and glial integrity: an exploration of associations of cytokines and kynurenine metabolites with symptoms and attention. Behav. Brain Funct. 6, 32 (10.1186/1744-9081-6-32) 20534153 PMC2900218

[53] Leffa DT, Torres ILS, Rohde LA 2018 A Review on the role of inflammation in attention-deficit/hyperactivity disorder. Neuroimmunomodulation 25, 328–333. (10.1159/000489635) 29874674

[54] He Y, Chen J, Zhu LH, Hua LL, Ke FF 2017 Maternal smoking during pregnancy and ADHD: results from a systematic review and meta-analysis of prospective cohort studies. J. Atten. Disord. 1087054717696766 (10.1177/1087054717696766) 29039728

[55] Freitag CM, Rohde LA, Lempp T, Romanos M 2010 Phenotypic and measurement influences on heritability estimates in childhood ADHD. Eur. Child Adolesc. Psychiatry. 19, 311–323. (10.1007/s00787-010-0097-5) 20213230

[56] Lange KW, Reichl S, Lange KM, Tucha L, Tucha O 2010 The history of attention deficit hyperactivity disorder. Atten. Defic. Hyperact Disord. 2, 241–255. (10.1007/s12402-010-0045-8) 21258430 PMC3000907

[57] Gauthier TW 2015 Prenatal alcohol exposure and the developing immune system. Alcohol Res. 37, 279–285. 26695750 10.35946/arcr.v37.2.11 PMC4590623

[58] Gauthier TW, Drews-Botsch C, Falek A, Coles C, Brown LA 2005 Maternal alcohol abuse and neonatal infection. Alcohol Clin. Exp. Res. 29, 1035–1043. (10.1097/01.ALC.0000167956.28160.5E) 15976530

[59] Prosser DE, Jones G 2004 Enzymes involved in the activation and inactivation of vitamin D. Trends Biochem. Sci. 29, 664–673. (10.1016/j.tibs.2004.10.005) 15544953

[60] Zhong W, Gu B, Gu Y, Groome LJ, Sun J, Wang Y 2014 Activation of vitamin D receptor promotes VEGF and CuZn-SOD expression in endothelial cells. J. Steroid Biochem. Mol. Biol. 140, 56–62. (10.1016/j.jsbmb.2013.11.017) 24316428 PMC3915503

[61] Pojednic RM, Ceglia L, Olsson K, Gustafsson T, Lichtenstein AH, Dawson-Hughes B, Fielding RA 2015 Effects of 1,25-dihydroxyvitamin D3 and vitamin D3 on the expression of the vitamin D receptor in human skeletal muscle cells. Calcif Tissue Int. 96, 256–263. (10.1007/s00223-014-9932-x) 25479835 PMC4429607

[62] Kato S 2000 The function of vitamin D receptor in vitamin D action. J. Biochem. 127, 717–722. (10.1093/oxfordjournals.jbchem.a022662) 10788778

[63] Gombart AF, Borregaard N, Koeffler HP 2005 Human cathelicidin antimicrobial peptide (CAMP) gene is a direct target of the vitamin D receptor and is strongly up-regulated in myeloid cells by 1,25-dihydroxyvitamin D3. FASEB J. 19, 1067–1077. (10.1096/fj.04-3284com) 15985530

[64] Wang TT et al 2004 Cutting edge: 1,25-dihydroxyvitamin D3 is a direct inducer of antimicrobial peptide gene expression. J. Immunol. 173, 2909–2912. (10.4049/jimmunol.173.5.2909) 15322146

[65] Wang Y, Becklund BR, DeLuca HF 2009 Identification of a highly specific and versatile vitamin D receptor antibody. Arch. Biochem. Biophys. 494, 166–177. (10.1016/j.abb.2009.11.029) 19951695

[66] Bouillon R et al 2008 Vitamin D and human health: lessons from vitamin D receptor null mice. Endocr. Rev. 29, 726–776. (10.1210/er.2008-0004) 18694980 PMC2583388

[67] Carlberg C, Seuter S 2009 A genomic perspective on vitamin D signaling. Anticancer Res. 29, 3485–3493. 19667142

[68] Berridge MJ 2018 Vitamin D deficiency: infertility and neurodevelopmental diseases (attention deficit hyperactivity disorder, autism, and schizophrenia). Am. J .Physiol. Cell Physiol. 314, C135–CC51. (10.1152/ajpcell.00188.2017) 29070492

[69] Jin D, Wu S, Zhang YG, Lu R, Xia Y, Dong H, Sun J 2015 Lack of vitamin D receptor causes dysbiosis and changes the functions of the murine intestinal microbiome. Clin. Ther. 37, 996–1009. (10.1016/j.clinthera.2015.04.004) 26046242

[70] Wu S et al 2015 Intestinal epithelial vitamin D receptor deletion leads to defective autophagy in colitis. Gut. 64, 1082–1094. (10.1136/gutjnl-2014-307436) 25080448 PMC4312277

[71] Zhang YG et al 2015 Tight junction CLDN2 gene is a direct target of the vitamin D receptor. Sci. Rep. 5, 10642 (10.1038/srep10642) 26212084 PMC4650691

[72] Wang J et al 2016 Genome-wide association analysis identifies variation in vitamin D receptor and other host factors influencing the gut microbiota. Nat. Genet. 48, 1396–1406. (10.1038/ng.3695) 27723756 PMC5626933

[73] García-Serna AM, Morales E In press. Neurodevelopmental effects of prenatal vitamin D in humans: systematic review and meta-analysis. Mol. Psychiatry. 10.1038/s41380-019-0357-9 30696940

[74] Vinkhuyzen AAE et al 2018 Gestational vitamin D deficiency and autism-related traits: the Generation R Study. Mol. Psychiatry. 23, 240–246. (10.1038/mp.2016.213) 27895322 PMC5554617

[75] Veena SR et al 2017 Association between maternal vitamin D status during pregnancy and offspring cognitive function during childhood and adolescence. Asia Pac. J. Clin. Nutr. 26, 438–449. 28429909 10.6133/apjcn.032016.07 PMC5965666

[76] Kaushal M, Magon N 2013 Vitamin D in pregnancy: a metabolic outlook. Indian J. Endocrinol. Metab. 17, 76–82. (10.4103/2230-8210.107862) 23776856 PMC3659910

[77] Risch N, Hoffmann TJ, Anderson M, Croen LA, Grether JK, Windham GC 2014 Familial recurrence of autism spectrum disorder: evaluating genetic and environmental contributions. Am. J. Psychiatry. 171, 1206–1213. (10.1176/appi.ajp.2014.13101359) 24969362

[78] Feng J et al 2017 Clinical improvement following vitamin D3 supplementation in Autism Spectrum Disorder. Nutr. Neurosci. 20, 284–290. (10.1080/1028415X.2015.1123847) 26783092

[79] Jia F, Wang B, Shan L, Xu Z, Staal WG, Du L. 2015 Core symptoms of autism improved after vitamin D supplementation. Pediatrics. 135, e196–e198. (10.1542/peds.2014-2121) 25511123

[80] Coşkun S, Şimşek Ş, Camkurt MA, Çim A, Çelik SB 2016 Association of polymorphisms in the vitamin D receptor gene and serum 25-hydroxyvitamin D levels in children with autism spectrum disorder. Gene. 588, 109–114. (10.1016/j.gene.2016.05.004) 27155524

[81] Saad K et al 2016 Vitamin D status in autism spectrum disorders and the efficacy of vitamin D supplementation in autistic children. Nutr. Neurosci. 19, 346–351. (10.1179/1476830515Y.0000000019) 25876214

[82] Wang T, Shan L, Du L, Feng J, Xu Z, Staal WG, Jia F 2016 Serum concentration of 25-hydroxyvitamin D in autism spectrum disorder: a systematic review and meta-analysis. Eur. Child Adolesc. Psychiatry. 25, 341–350. (10.1007/s00787-015-0786-1) 26514973

[83] Daraki V et al 2018 High maternal vitamin D levels in early pregnancy may protect against behavioral difficulties at preschool age: the Rhea mother-child cohort, Crete, Greece. Eur. Child Adolesc. Psychiatry. 27, 79–88. (10.1007/s00787-017-1023-x) 28685401

[84] Morales E et al 2015 Vitamin D in pregnancy and attention deficit hyperactivity disorder-like symptoms in childhood. Epidemiology. 26, 458–465. (10.1097/EDE.0000000000000292) 25867115

[85] Sahin N, Altun H, Kurutas EB, Balkan D 2018 Vitamin D and vitamin D receptor levels in children with attention-deficit/hyperactivity disorder. Neuropsychiatr Dis. Treat. 14, 581–585. (10.2147/NDT.S158228) 29497301 PMC5822841

[86] Strøm M et al 2014 Vitamin D measured in maternal serum and offspring neurodevelopmental outcomes: a prospective study with long-term follow-up. Ann. Nutr. Metab. 64, 254–261. (10.1159/000365030) 25300268

[87] Gustafsson P et al 2015 Vitamin D status at birth and future risk of attention deficit/hyperactivity disorder (ADHD). PLoS ONE. 10, e0140164 (10.1371/journal.pone.0140164) 26509435 PMC4624803

[88] Khoshbakht Y, Bidaki R, Salehi-Abargouei A 2018 Vitamin D status and attention deficit hyperactivity disorder: a systematic review and meta-analysis of observational studies. Adv. Nutr. 9, 9–20. (10.1093/advances/nmx002) 29438455 PMC6333940

[89] Soto M, Herzog C, Pacheco JA, Fujisaka S, Bullock K, Clish CB, Kahn CR 2018 Gut microbiota modulate neurobehavior through changes in brain insulin sensitivity and metabolism. Mol.Psychiatry. 23, 2287–2301. (10.1038/s41380-018-0086-5) 29910467 PMC6294739

[90] Patrick RP, Ames BN 2014 Vitamin D hormone regulates serotonin synthesis. Part 1: relevance for autism. FASEB J. 28, 2398–2413. (10.1096/fj.13-246546) 24558199

[91] Chugani DC, Muzik O, Rothermel R, Behen M, Chakraborty P, Mangner T, Da Silva E, Chugani HT 1997 Altered serotonin synthesis in the dentatothalamocortical pathway in autistic boys. Ann. Neurol. 42, 666–669. (10.1002/ana.410420420) 9382481

[92] Kaneko I et al 2015 1,25-Dihydroxyvitamin D regulates expression of the tryptophan hydroxylase 2 and leptin genes: implication for behavioral influences of vitamin D. FASEB J. 29, 4023–4035. (10.1096/fj.14-269811) 26071405

[93] Jiang P et al 2014 Neurochemical effects of chronic administration of calcitriol in rats. Nutrients. 6, 6048–6059. (10.3390/nu6126048) 25533012 PMC4277014

[94] Sabir MS et al 2018 Optimal vitamin D spurs serotonin: 1,25-dihydroxyvitamin D represses serotonin reuptake transport. Genes Nutr. 13, 19 (10.1186/s12263-018-0605-7) 30008960 PMC6042449

[95] Bach H, Arango V, Huang YY, Leong S, Mann JJ, Underwood MD 2011 Neuronal tryptophan hydroxylase expression in BALB/cJ and C57Bl/6 J mice. J. Neurochem. 118, 1067–1074. (10.1111/j.1471-4159.2011.07379.x) 21740442 PMC4222035

[96] Russo AM et al 2019 Social approach, anxiety, and altered tryptophan hydroxylase 2 activity in juvenile BALB/c and C57BL/6 J mice. Behav. Brain Res. 359, 918–926. (10.1016/j.bbr.2018.06.019) 29935278

[97] Kane MJ, Angoa-Peréz M, Briggs DI, Sykes CE, Francescutti DM, Rosenberg DR, Kuhn DM 2012 Mice genetically depleted of brain serotonin display social impairments, communication deficits and repetitive behaviors: possible relevance to autism. PLoS ONE 7, e48975 (10.1371/journal.pone.0048975) 23139830 PMC3490915

[98] Li ZS, Pham TD, Tamir H, Chen JJ, Gershon MD 2004 Enteric dopaminergic neurons: definition, developmental lineage, and effects of extrinsic denervation. J. Neurosci. 24, 1330–1339. (10.1523/JNEUROSCI.3982-03.2004) 14960604 PMC6730344

[99] Dussik CM et al 2018 Gene expression profiling and assessment of vitamin D and serotonin pathway variations in patients with irritable bowel syndrome. J. Neurogastroenterol. Motil. 24, 96–106. (10.5056/jnm17021) 29291611 PMC5753908

[100] Kwon YH et al 2019 Modulation of gut microbiota composition by serotonin signaling influences intestinal immune response and susceptibility to colitis. Cell Mol. Gastroenterol. Hepatol. 7, 709–728. (10.1016/j.jcmgh.2019.01.004) 30716420 PMC6462823

[101] Patrick RP, Ames BN 2015 Vitamin D and the ω-3 fatty acids control serotonin synthesis and action, part 2: relevance for ADHD, bipolar disorder, schizophrenia, and impulsive behavior. FASEB J. 29, 2207–2222. (10.1096/fj.14-268342) 25713056

[102] Orme RP, Bhangal MS, Fricker RA 2013 Calcitriol imparts neuroprotection in vitro to midbrain dopaminergic neurons by upregulating GDNF expression. PLoS ONE 8, e62040 (10.1371/journal.pone.0062040) 23626767 PMC3633905

[103] Van Ameringen M et al. 2019 The gut microbiome in psychiatry: a primer for clinicians. Depress Anxiety. 10.1002/da.22936 31356715

[104] Hovde MJ, Larson GH, Vaughan RA, Foster JD 2019 Model systems for analysis of dopamine transporter function and regulation. Neurochem. Int. 123, 13–21. (10.1016/j.neuint.2018.08.015) 30179648 PMC6338519

[105] Vles JS, Feron FJ, Hendriksen JG, Jolles J, van Kroonenburgh MJ, Weber WE. 2003 Methylphenidate down-regulates the dopamine receptor and transporter system in children with attention deficit hyperkinetic disorder (ADHD). Neuropediatrics. 34, 77–80. (10.1055/s-2003-39602) 12776228

[106] Kesby JP, Cui X, O'Loan J, McGrath JJ, Burne TH, Eyles DW 2010 Developmental vitamin D deficiency alters dopamine-mediated behaviors and dopamine transporter function in adult female rats. Psychopharmacology 208, 159–168. (10.1007/s00213-009-1717-y) 19921153

[107] Dehbokri N, Noorazar G, Ghaffari A, Mehdizadeh G, Sarbakhsh P, Ghaffary S 2019 Effect of vitamin D treatment in children with attention-deficit hyperactivity disorder. World J. Pediatr. 15, 78–84. (10.1007/s12519-018-0209-8) 30456564

[108] Seyedi M et al 2019 The effect of vitamin D3 supplementation on Serum BDNF, dopamine, and serotonin in children with attention-deficit/hyperactivity disorder. CNS Neurol. Disord. Drug Targets. 18, 496–501. (10.2174/1871527318666190703103709) 31269890

[109] Sigurdardottir HL et al 2016 Effects of norepinephrine transporter gene variants on NET binding in ADHD and healthy controls investigated by PET. Hum. Brain Mapp. 37, 884–895. (10.1002/hbm.23071) 26678348 PMC4949568

[110] Eiden LE, Weihe E 2011 VMAT2: a dynamic regulator of brain monoaminergic neuronal function interacting with drugs of abuse. Ann. NY Acad. Sci. 1216, 86–98. (10.1111/j.1749-6632.2010.05906.x) 21272013 PMC4183197

[111] Chandler DJ, Waterhouse BD, Gao WJ 2014 New perspectives on catecholaminergic regulation of executive circuits: evidence for independent modulation of prefrontal functions by midbrain dopaminergic and noradrenergic neurons. Front. Neural Circuits. 8, 53 (10.3389/fncir.2014.00053) 24904299 PMC4033238

[112] Gonon F 2009 The dopaminergic hypothesis of attention-deficit/hyperactivity disorder needs re-examining. Trends Neurosci. 32, 2–8. (10.1016/j.tins.2008.09.010) 18986716

[113] Reale L, Bartoli B, Cartabia M, Zanetti M, Costantino MA, Canevini MP, Termine C, Bonati M 2017 Comorbidity prevalence and treatment outcome in children and adolescents with ADHD. Eur. Child Adolesc. Psychiatry. 26, 1443–1457. (10.1007/s00787-017-1005-z) 28527021

[114] Eyles DW, Feron F, Cui X, Kesby JP, Harms LH, Ko P, Mcgrath JJ, Burne THJ 2009 Developmental vitamin D deficiency causes abnormal brain development. Psychoneuroendocrinology. 34(Suppl. 1), S247–S257. (10.1016/j.psyneuen.2009.04.015) 19500914

[115] Pertile RA, Cui X, Eyles DW 2016 Vitamin D signaling and the differentiation of developing dopamine systems. Neuroscience. 333, 193–203. (10.1016/j.neuroscience.2016.07.020) 27450565

[116] Cui X, Pelekanos M, Liu PY, Burne TH, McGrath JJ, Eyles DW 2013 The vitamin D receptor in dopamine neurons; its presence in human substantia nigra and its ontogenesis in rat midbrain. Neuroscience. 236, 77–87. (10.1016/j.neuroscience.2013.01.035) 23352937

[117] van de Wouw M et al 2018 Short-chain fatty acids: microbial metabolites that alleviate stress-induced brain-gut axis alterations. J. Physiol. 596, 4923–4944. (10.1113/JP276431) 30066368 PMC6187046

[118] Nguyen TL, Vieira-Silva S, Liston A, Raes J 2015 How informative is the mouse for human gut microbiota research? Dis. Model Mech. 8, 1–16. (10.1242/dmm.017400) 25561744 PMC4283646

[119] Adams JB, Johansen LJ, Powell LD, Quig D, Rubin RA 2011 Gastrointestinal flora and gastrointestinal status in children with autism–comparisons to typical children and correlation with autism severity. BMC Gastroenterol. 11, 22 (10.1186/1471-230X-11-22) 21410934 PMC3072352

[120] Strati F et al 2017 New evidences on the altered gut microbiota in autism spectrum disorders. Microbiome. 5, 24 (10.1186/s40168-017-0242-1) 28222761 PMC5320696

[121] MacFabe DF, Cain NE, Boon F, Ossenkopp KP, Cain DP 2011 Effects of the enteric bacterial metabolic product propionic acid on object-directed behavior, social behavior, cognition, and neuroinflammation in adolescent rats: Relevance to autism spectrum disorder. Behav. Brain Res. 217, 47–54. (10.1016/j.bbr.2010.10.005) 20937326

[122] Hoyles L, Snelling T, Umlai UK, Nicholson JK, Carding SR, Glen RC, Mcarthur S 2018 Microbiome-host systems interactions: protective effects of propionate upon the blood-brain barrier. Microbiome. 6, 55 (10.1186/s40168-018-0439-y) 29562936 PMC5863458

[123] Stilling RM, van de Wouw M, Clarke G, Stanton C, Dinan TG, Cryan JF. 2016 The neuropharmacology of butyrate: The bread and butter of the microbiota-gut-brain axis? Neurochem. Int. 99, 110–132. (10.1016/j.neuint.2016.06.011) 27346602

[124] Sharon G et al 2019 Human gut microbiota from autism spectrum disorder promote behavioral symptoms in mice. Cell 177, 1600–1618. (10.1016/j.cell.2019.05.004) 31150625 PMC6993574

[125] Kilb W, Fukuda A 2017 Taurine as an essential neuromodulator during perinatal cortical development. Front. Cell Neurosci. 11, 328 (10.3389/fncel.2017.00328) 29123472 PMC5662885

[126] Dhaher R, Damisah EC, Wang H, Gruenbaum SE, Ong C, Zaveri HP, Gruenbaum BF, Eid T 2014 5-aminovaleric acid suppresses the development of severe seizures in the methionine sulfoximine model of mesial temporal lobe epilepsy. Neurobiol. Dis. 67, 18–23. (10.1016/j.nbd.2014.03.006) 24632421 PMC4035438

[127] Wang L, Christophersen CT, Sorich MJ, Gerber JP, Angley MT, Conlon MA 2012 Elevated fecal short chain fatty acid and ammonia concentrations in children with autism spectrum disorder. Dig. Dis. Sci. 57, 2096–2102. (10.1007/s10620-012-2167-7) 22535281

[128] Kang DW et al 2018 Differences in fecal microbial metabolites and microbiota of children with autism spectrum disorders. Anaerobe. 49, 121–131. (10.1016/j.anaerobe.2017.12.007) 29274915

[129] Chen JS 1995 Alcohol dehydrogenase: multiplicity and relatedness in the solvent-producing clostridia. FEMS Microbiol. Rev. 17, 263–273. (10.1111/j.1574-6976.1995.tb00210.x) 7576768

[130] George HA, Johnson JL, Moore WE, Holdeman LV, Chen JS 1983 Acetone, isopropanol, and butanol production by *Clostridium beijerinckii* (syn. *Clostridium butylicum*) and *Clostridium aurantibutyricum*. Appl. Environ. Microbiol. 45, 1160–1163. (10.1128/AEM.45.3.1160-1163.1983) 16346237 PMC242427

[131] Kraut JA, Kurtz I 2008 Toxic alcohol ingestions: clinical features, diagnosis, and management. Clin. J. Am Soc. Nephrol. 3, 208–225. (10.2215/CJN.03220807) 18045860

[132] Abramson S, Singh AK 2000 Treatment of the alcohol intoxications: ethylene glycol, methanol and isopropanol. Curr. Opin. Nephrol. Hypertens. 9, 695–701. (10.1097/00041552-200011000-00017) 11128434

[133] Liebisch G et al 2019 Quantification of fecal short chain fatty acids by liquid chromatography tandem mass spectrometry-investigation of pre-analytic stability. Biomolecules 9, 121 (10.3390/biom9040121) PMC6523859 30925749

[134] Liu S et al 2019 Altered gut microbiota and short chain fatty acids in Chinese children with autism spectrum disorder. Sci. Rep. 9, 287 (10.1038/s41598-018-36430-z) 30670726 PMC6342986

[135] Wang Y et al 2020 Probiotics and fructo-oligosaccharide intervention modulate the microbiota-gut brain axis to improve autism spectrum reducing also the hyper-serotonergic state and the dopamine metabolism disorder. Pharmacol. Res. 157,104784 (10.1016/j.phrs.2020.104784) 32305492

[136] Shultz SR et al 2008 Intracerebroventricular injection of propionic acid, an enteric bacterial metabolic end-product, impairs social behavior in the rat: implications for an animal model of autism. Neuropharmacology 54, 901–911. (10.1016/j.neuropharm.2008.01.013) 18395759

[137] Witters P et al 2016 Autism in patients with propionic acidemia. Mol. Genet. Metab. 119, 317–321. (10.1016/j.ymgme.2016.10.009) 27825584

[138] de la Batie CD et al 2018 Autism spectrum disorders in propionic acidemia patients. J. Inherit. Metab. Dis. 41, 623–629. (10.1007/s10545-017-0070-2) 28856627

[139] Al-Owain M et al 2013 Autism spectrum disorder in a child with propionic acidemia. JIMD Rep. 7, 63–66. (10.1007/8904_2012_143) 23430497 PMC3573175

[140] Maes M, Mihaylova I, Ruyter MD, Kubera M, Bosmans E 2007 The immune effects of TRYCATs (tryptophan catabolites along the IDO pathway): relevance for depression and other conditions characterized by tryptophan depletion induced by inflammation. Neuro. Endocrinol. Lett. 28, 826–831. 18063923

[141] Aarsland TI, Landaas ET, Hegvik TA, Ulvik A, Halmøy A, Ueland PM, Haavik J 2015 Serum concentrations of kynurenines in adult patients with attention-deficit hyperactivity disorder (ADHD): a case-control study. Behav. Brain Funct. 11, 36 (10.1186/s12993-015-0080-x) 26542774 PMC4636001

[142] Evangelisti M et al 2017 Changes in serum levels of kynurenine metabolites in paediatric patients affected by ADHD. Eur. Child Adolesc. Psychiatry. 26, 1433–1441. (10.1007/s00787-017-1002-2) 28527020

[143] Sumitomo A et al 2018 Methylphenidate and guanfacine ameliorate ADHD-like phenotypes in *Fez1*-deficient mice. Mol. Neuropsychiatry. 3, 223–233. (10.1159/000488081) 29888233 PMC5981631

[144] Chatterjee I, Lu R, Zhang Y, Zhang J, Dai Y, Xia Y, Sun J 2020 Vitamin D receptor promotes healthy microbial metabolites and microbiome. Sci. Rep. 10, 7340 (10.1038/s41598-020-64226-7) 32355205 PMC7192915

